# Microsatellite instability in patients with chronic B-cell lymphocytic leukaemia

**DOI:** 10.1038/sj.bjc.6602532

**Published:** 2005-04-05

**Authors:** E Niv, Y Bomstein, M Yuklea, M Lishner

**Affiliations:** 1 Department of Medicine, Meir Hospital, Kfar-Saba, Israel; 2 Department of Hematology, Meir Hospital, Kfar-Saba, Israel; 3 Department of Oncogenetic Laboratory, Meir Hospital, Kfar-Saba, Israel; 4 Sackler Faculty of Medicine, Tel-Aviv University, Israel

**Keywords:** microsatellite instability (MSI), loss of heterozygosity (LOH), chronic B-cell lymphocytic leukaemia (B-CLL), replication errors positive phenotype (RER+)

## Abstract

The purpose of our study was to evaluate the microsatellite instability (MSI) at selected loci with known involvement in the oncogenesis of chronic B-cell lymphocytic leukaemia (B-CLL). DNA from B cells (tumour cells) and from T cells (normal controls) of 27 samples of 26 patients with previously untreated B-CLL was extracted. Microsatellite instability in six microsatellite markers was tested using GeneScan Analysis Software. The rate of replication errors positive phenotype (RER+) was determined (MSI in more than 30% of examined loci). RER+ was found in four out of 27 paients (14.8%). A larger proportion of patients with stage C B-CLL exhibited RER+ than those with stage A or B (*P*<0.05). A higher prevalence of RER+ was demonstrated in a subgroup of patients with additional malignancies (three out of eight patients) in comparison with patients with B-CLL alone (1/19) (*P*=0.031). In conclusion, our study demonstrated that MSI might have a more prominent role in pathogenesis of B-CLL than reported todate. This may result from a selection of microsatellite markers adjacent to chromosomal loci, which are involved in B-cell malignancies, and using GeneScan Analysis Software, which is most modern and precise method of microsatellite analysis.

Chronic B-cell lymphocytic leukaemia (B-CLL) is the most common leukaemia in the Western world. It is characterised by the accumulation of long-lived, functionally inactive, mature appearing neoplastic B lymphocytes. Although several studies demonstrated a role of trisomy 12 and structural abnormalities of chromosome 6, 11, 13 and 14 in the pathogenesis of B-CLL, the molecular mechanisms involved in this relatively common disease are still poorly understood (Montserrat *et al*, 1997; Fundia *et al*, 1998; Dohner *et al*, 2000). Recently, a possible role of DNA mismatch repair defects and microsatellite instability (MSI) in the pathogenesis of CLL has been debated (Gartenhaus *et al*, 1996; Pabst *et al*, 1996; Volpe *et al*, 1996; Sanz-Vaque *et al*, 2001).

Microsatellites are short repeat sequences dispersed throughout the genome. They are composed of mono-, di-, tri-, or tetranucleotide repeats. Microsatellites are highly unstable, that is, the number of repeat units tends to change due to slippage errors during DNA replications. These errors are supposedly corrected by DNA repair enzymes, like errors in any different part of the genome. Cells with alteration in DNA mismatch repair enzymes are not able to repair correctly errors during DNA replication, demonstrating RER+ (replication errors positive phenotype). Being highly unstable makes microsatellites the best markers of RER+. In other words, while length alterations of microsatellite sequences are usually phenotypically silent, they reflect a general defect in the DNA repair mechanism. Comparison of length of microsatellites of malignant cells to that of normal cells enables to find the mutations inside the microsatellites and to determine a malfunction of DNA mismatch repair enzymes. Inactivation of mismatch repair genes and progressive accumulation of replication errors is one of the molecular pathways of oncogenesis.

Microsatellite instability has been found in up to 90% of tumours of the hereditary nonpolyposis colorectal cancer because of germ-line mutations and further damage to the second allele within the specific mismatch repair genes *hMSH1, hMLH2* and *hPMS2* (Peltomaki and Vasen, 1997; Boland *et al*, 1998). Microsatellite instability is also a distinctive feature in nearly 15–20% of sporadic colorectal tumours (Peltomaki and Vasen, 1997; Boland *et al*, 1998; Toft and Arends, 1998; Samowitz *et al*, 1999; Stone *et al*, 2000).

Recently, a possible role of abnormalities of DNA repair and MSI in the pathogenesis of haematological malignancies, like Hodgkin's disease, non-Hodgkin's lymphoma, B-cell CLL, Richter's syndrome, hairy cell leukaemia, etc., has been debated (Gartenhaus *et al*, 1996; Pabst *et al*, 1996; Randerson *et al*, 1996; Tasak *et al*, 1996; Volpe *et al*, 1996; Mark *et al*, 1998; Rimsza *et al*, 2000; Sanz-Vaque *et al*, 2001). Most previous studies examined genomic instability in a wide variety of genomic loci, which were arbitrarily selected and were known to be unstable in solid tumours. The purpose of our study was to evaluate the instability at microsatellite markers adjacent to chromosomal loci, which are known to be involved in development of B-cell neoplasms, including of B-CLL, or at loci that encode DNA mismatch repair enzymes.

## MATERIALS AND METHODS

### Case selection

Consecutive patients, with previously untreated B-CLL were recruited from the Department of Hematology of Meir hospital between April 2000 and April 2001. The study group was composed of all newly diagnosed B-CLL patients during 2000–2001 and of previouly untreated patients with known B-CLL, who came to the rutine follow-up visit during this year. B-cell lymphocytic leukaemia was diagnosed according to the standard criteria. Demographic, laboratory, as well as clinical data, were collected. The medical records of all the participants were checked for additional malignancies in the past. Patients who received chemotherapy for other malignancies were excluded. Surgical treatment was not considered as an exclusion criteria.

The study was approved by the local research ethics committee. All patients provided an informed consent.

### DNA collection and processing

Peripheral blood samples were collected from the patients with CLL and mononuclear cells were isolated. B cells were separated from T cells by negative selection of B cells using magnetic beads coated with anti-CD3 antibodies and negative selection of T cells using anti-CD19 (Dynal AS, Oslo, Norway). To verify the purity of these two cell populations, flow cytometric analysis was used.

The isolated B cells were considered neoplastic. Patients' own T cells were chosen as the normal, negative controls, similar to previous publications (Gartenhaus *et al*, 1996). DNA was extracted from B and T cells (tumour and normal cells, respectively) using Puregene kit (Gentra Systems, Minneapolis, USA), according to the manufacturer's instructions.

### Microsatellite marker analysis

Primer sequences at six different loci were selected from Genome Database (Table 1). *P16, MLL* and *Leu1* are known to be involved in haematological tumours: (1) *P16* is a tumour suppressor gene, which encodes an inhibitor of cyclin-dependent kinase 4 (CDK4). Binding of the product of *P16* to CDK4 prevents progression through the cell cycle. Alterations of the *P16* gene, like deletions, hypermethylation and mutations were reported in 30% of transformed variants of non-Hodgkin's lymphoma (NHL). (2) *11q21–23* is involved in translocations that are very common in AML and ALL. One of the genes described in *11q23* is MLL, which is rearranged with a variety of partners in haematological malignancies (Takeuchi *et al*, 1997, ; Webb *et al*, 1999). Deletions in *11q23* were also observed in B-CLL (Dohner *et al*, 2000). (3) Structural abnormalities in *13q14* are very frequent in B-CLL (Fundia *et al*, 1998; Dohner *et al*, 2000). *Rb1* is located in this region and was found to be involved in tumorigenesis. Recently, two novel candidate tumour suppressor genes *Leu1* and *Leu2* were mapped to this region.

In addition, *hMLH-1, hMSH-2* and *APC* were chosen. *hMLH-1* and *hMSH-2* encode DNA mismatch repair enzymes and are involved in both haematological and solid malignancies. The *APC* gene was selected mainly as a control. Despite possible involvement of *APC* gene in tumorigenesis of MALT lymphoma and gastric high-grade large B-cell lymphoma (Calvert *et al*, 1995; Starostik *et al*, 2000), this gene has no known role in the pathogenesis of this tumour.

The microsatellites within or closely located to the above loci were chosen (sequences are presented in Table 1). One end of each primer was synthesised and labelled by FAM (Mycrosynth, Balgach, Switzerland) and the opposite was synthesised by Sigma-Genosys (Cambridgeshire, UK). Microsatellite loci in genomic DNA were amplified by polymerase chain reaction (PCR) using Biometra Thermocycler (Whatman, Gottingen, Germany) in 15 *μ*l volume. The PCR mixture consisted of 1 × PCR buffer (10 mM Tris-HCL, pH 8.3, 50 mM KCL, 1.5 mM MgCl_2_), 0.2 mM of each dNTP (Roche, Mannheim, Germany), 6 pmol of each primer and 1.5 units of *Taq* polymerase (Sigma, MO, USA). Both tumour and normal DNA were subjected to 36 cycles of PCR with automated temperature cycling programme as follows: denaturation at 94°C for 30 s, annealing at 55°C for all primers except P16 (57.5°C) for 30 s and elongation at 72°C for 30 s. Amplification was concluded with extension at 72°C for 30 min to avoid incorrect allele cells due to tendency of *Taq* DNA polymerase to add A base to 3′ end of DNA. This long extension promotes A addition to all the PCR products.

Fluorescent PCR products were subjected to electrophoresis on denaturing polyacrylamide gel and fractionated by Automated Fluorescent DNA Sequencer (ABI 377, PE Biosystems). The data were processed using GeneScan Analysis Software (Perkin Elmer, Foster City, CA, USA).

**Figure 1:**
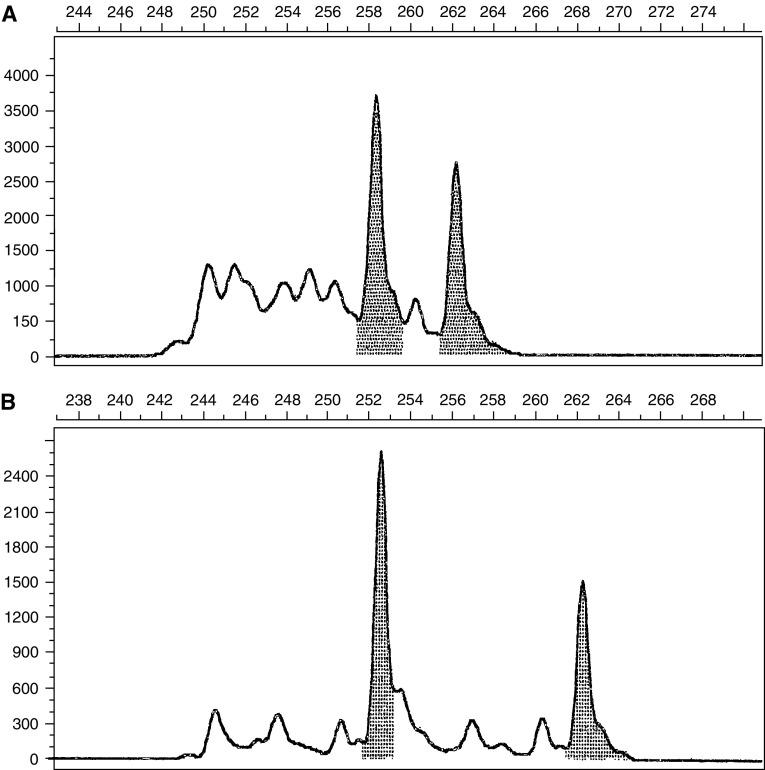
Representative allelic profile of microsatellite instability at *hMLH1* locus. (**A**) T cells (normal cells). Horizontal – base pairs scale, vertical – fluorescence scale. In the normal cells, the two major peaks are 258 and 262 base pairs long representing the two alleles of this microsatellite. (**B**) B cells (malignant cells). Horizontal – base pairs scale, vertical – fluorescence scale. In the malignant cells, the one of the alleles of the microsatellite is 262 base pairs long like in the normal cells, but the second one is 252 base pairs long (which is different from the allel of 258 base pairs).

**Figure 2:**
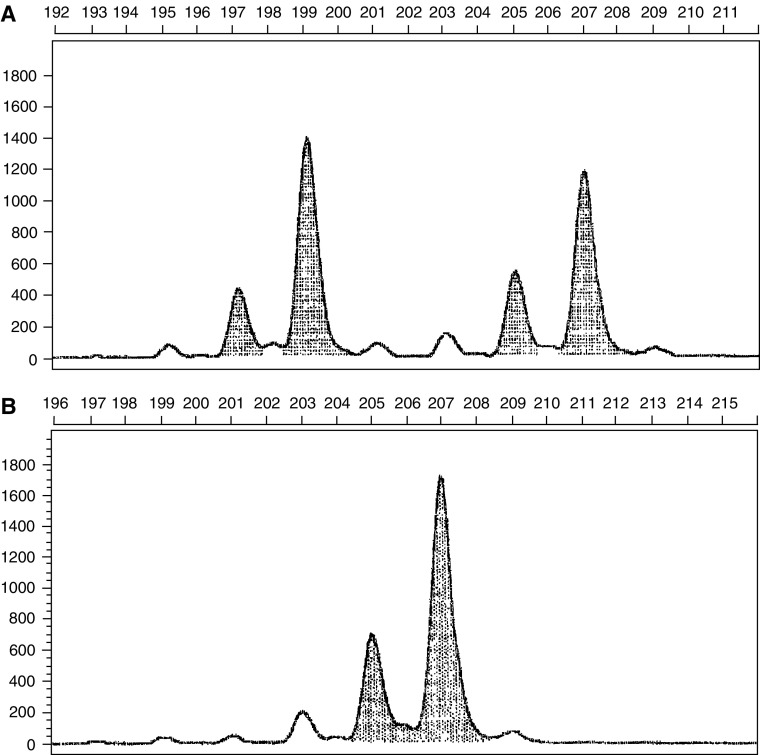
Representative allelic profile of loss of heterozygosity at *Leu1* locus. (**A**) T cells (normal cells). Horizontal – base pairs scale, vertical – fluorescence scale. In the normal cells, the two alleles of this microsatellite are 109 and 207 base pairs long. (**B**) B cells (malignant cells). Horizontal – base pairs scale, vertical – fluorescence scale. In the malignant cells the alleles of 109 disappeared and only the alleles of 207 base pairs remained.

We used the common acceptable definitions of MSI and loss of heterozygosity (LOH) (Dietmaier *et al*, 1997). Microsatellite instability was defined as a change of any length due to either insertion or deletion of repeating units, in a microsatellite within tumour cells (B cells) compared to normal cells (T cells). This was seen as novel peak/s in B cells DNA differing in size and location from T cells DNA (Figure 1). Unlike MSI, LOH was defined as loss of one of the pre-existing alleles in tumour cells compared to normal tissue (Figure 2). In this situation, one cannot easily discern whether this represents true LOH or MSI in which the shifted allele has comigrated with the remaining wild-type allele. These cases were defined as LOH, although scoring results in this fashion would appear to bias the data in favour of LOH group.

RER positivity was defined as the finding of MSI in more than 30% of examined loci, as it commonly accepted (Boland *et al*, 1998).

### Statistical analysis

Standard descriptive statistics, including means, standard deviations, ranges and frequency calculations were used to characterise the study group. For comparisons, a χ^2^ and Student's *t*-tests with two-sided type I error of 0.05 was used to assess statistical significance. In addition, a test for comparison of two proportions was used. *P* less than 0.05 with *z* more than 1.65 was considered as statistically significant.

## RESULTS

A total of 26 patients with previously untreated B-CLL participated in the study. Of them, 16 patients were newly diagnosed B-CLL patients and the rest were previously untreated B-CLL patients who were at follow-up in the Department of Hematology of Meir Hospital.

Patients' characteristics are presented in Table 2. The study group included 10 women and 16 men with a mean age of 69.7 years (range, 45–86 years) and a mean leucocyte count of 60 456/*μ*l. In all, 16 patients had stage A CLL, six had stage B and five stage C (according to Binet's classification). Samples 4 and 27 were collected from the same patient after progression from stage B to C. According to their medical records, eight patients had additional malignancies in the past (Table 2), but none received chemo or radiotherapy for these tumours. All eight patients were at complete remision from these tumours at the time of the study.

Table 3 presents the results of microsatellite markers' analysis of the study group. Four patients (Gartenhaus *et al*, 1996; Tasak *et al*, 1996; Mark *et al*, 1998; Duval and Hamelin, 2002a, 2002b) were found to be RER positive (14.8%). The mean age and the mean leucocyte count were not significantly different from the patients without RER positivity (*P*=0.1).

A higher prevalence of RER positivity was demonstrated in a subgroup of patients with additional malignancies in the past (3/8 RER positive patients) compared to patients without history of tumours (1/19 RER positive patients) (*P*=0.031). No correlation was found between the prevalence of RER+ and the type of additional malignancy. Similarly, RER positivity was more common at stage C (40%), compared to stages A or B (12.5 and 0% respectively) (*P*<0.05). There was no difference in the rate of RER positivity between stages A and B.

Table 3 highlights additional interesting observations. First, the progression of instability from stage B (sample 4) to stage C (sample 27). Second, the marked instability of samples 14 and 15. These patients represented the MSI-H phenotype (instability at more than 40% of the loci examined). These patients also had a positive association with additional malignancies.

The frequency of MSI in each microsatellite locus was evaluated. Microsatellite instability was found at *Leu1* locus in three out of 27 (11.1%) of samples, at *MLL* in six out of 27 (22.2%), at *APC* in three out of 27 (11.1%), at *MSH2* in three out 27 (11.1%), at *P16* in three out 27 (11.1%), at *MLH1* in four out 27 (14.8%). In general, the rate of MSI at the examined loci was quite similar. Microsatellite instability in MLL locus was a little higher than in other loci, but it had no statistical significance (*P*=0.47). *APC* locus, which is considered to be unstable mostly in solid tumours, had a similar rate of instability to other loci in the current study.

## DISCUSSION

The present study has some unique features since we tested a relatively big group of previously untreated CLL for MSI in specific loci, which are involved in the pathogenesis of B-cell malignancies or encode for DNA mismatch repair enzymes. We also applied the GeneScan Analysis Software, which is considered the most precise method of microsatellite's analysis. We found RER-positivity rate of 14.8% in B-CLL. A significantly larger proportion of patients with stage C exhibited RER positivity than those with stages A or B. Also higher prevalence of RER positivity was demonstrated a group of patients with additional malignancies in the past. The frequency of MSI at different loci was similar.

Previous studies on MSI in B-CLL reported much lower prevalence of RER positivity. For example, Sanz-Vaque *et al* (2001) found MSI-low in 3/24 (13%) cases with B-CLL and no RER+ at all. Gartenhaus *et al* (1996) identified a mutator phenotype in 7% (2/29) of the cases studied. Volpe *et al* (1996) also determined very low frequency of MSI among chronic lymphoproliferative disorders. An analysis of these studies reveals that the MSI was examined in wide variety of genomic loci, which were arbitrarily selected. In addition, loci tested in these studies are known to be unstable in solid but not in haematological malignancies.

A study of special interest is the study of Novak *et al* (2002), which performed an analysis of 400 microsatellite markers for instability in 46 patients with B-CLL. They found 41 novel allels in 22 patients (range 1–22 markers per patient). These results suggest very low frequency of MSI in B-CLL. However, the examined loci were selected arbitrarily; the study group included previously treated by chemotherapy along with untreated patients. In addition, the analysis of MSI was performed by comparison of mononuclear with polymorphonuclear cells, which were considered as tumour and normal cells respectively, presuming that most of mononuclear cells are B cells. Gartenhaus *et al* (1996) demonstrated that B and T cells are different genetically and that comparison of PCR products of microsatellite loci allows to diagnose MSI. For this reason, in our study a separation of these two kinds of cells was performed.

Only one study examined MSI at chromosomal breakpoint cluster regions specific to haematological malignancies (Pabst *et al*, 1996). In contrast to our findings, they found a low frequency of MSI: of 36 patients with B-CLL only one had RER+ (2.8%). The higher rate of genomic instability and RER positivity in our study probably reflects the careful selection of a homogenous group of previously untreated CLL patients, meticulous selection of the tested loci and the use of very sensitive and specific modern method of Genescan Analysis for MSI detection.

The finding of higher frequency of instability in some microsatellite loci than in other loci is not surprising. This was already demonstrated on the model of hereditary nonpolyposis colorectal cancer. A list of hundreds of microsatellites was tested and a panel of five microsatellites was chosen as those with most common instability (Boland *et al*, 1998). Thus, an individual panel of microsatellites relevant to each malignancy should be composed and proximity to genomic loci, which are involved in the pathogenesis of specific tumours, should be considered.

Our assumption about higher rate of MSI in meticulously selected genetic loci received a strong support recently by ‘Real Common Target genes model’ (Duval *et al*, 2002; Duval and Hamelin, 2002a, 2002b; Woerner *et al*, 2003). According to this model, sets of few specific genes with high rate of mutations in different mismatch repair-deficient human cancers were identified. So far, this model was constructed only for colorectal, gastric and endometrial carcinomas. In order to develop such models for other tumours, data about MSI in different genetic loci of different tumours must be collected.

As mentioned above, the frequency of MSI in different selected loci in our study was quite similar. This is explained by meticulous selection of the tested loci with high probability of instability. However, surprisingly, the frequency of MSI in microsatellite near *APC* gene, which was selected mainly as a control, was high too. This finding may be explained by limited existing data about MSI in this gene. *APC* gene itself contains no microsatellite. In most previous studies about MSI in B-CLL, *APC* gene was not selected at all or different closely located microsatellites were tested.

Another important issue is an increase of instability upon the progression of CLL as was demonstrated by much higher rate of RER in stage C in comparison with stages A or B and an increase of instability in a patient upon disease progression. This finding is supported by a recently published study of Fulop *et al* (2003), who reported a high rate of MSI upon Richter's transformation of patients with B-CLL. It suggests that a defect in the DNA mismatch repair mechanism and progressive accumulation of replication errors have an important role in tumour biology of B-CLL. Unfortunately, the number of patients with advanced stage in our study group was small, because only previously untreated patients were included. In addition, the study period was relatively short for B-CLL (1 year) and only one patient progressed to the higher stage. Thus, large long-term prospective studies are needed to evaluate the correlation between MSI status and clinical course of B-CLL.

Additional prominent finding in the study is a high prevalence of RER positivity in group of patients with additional malignancies in the past. This cannot be explained by the effect of chemotherapy, since our patients were untreated. This phenomenon was also demonstrated by Ericson *et al* (2003) on basis of MSI analysis in individuals with multiple primary malignancies. In this study, MSI was identified in 63/154 (41%) tumours with a MSI-high pattern in 59 tumours. Immunohistochemical staining for DNA mismatch repair enzymes (*MLH1* and *MSH2*) in the examined tumours demonstrated a very high frequency of expression loss. Thus, the phenomenon of development multiple tumours during lifetime may reflect a profound defect in DNA mismatch repair mechanism.

In summary, our study demonstrated that MSI might have a more prominent role in the pathogenesis of B-CLL than that has been reported. This may result from a selection of microsatellite markers adjacent to chromosomal loci, which are involved in B-cell malignancies. These findings support the ‘Real Common Target genes’ theory of high MSI in specific genes that are involved in specific tumours. Additional important observations were the trend for more instability with the progression of B-CLL and the high rate of RER positivity in patients with additional tumours in the past.

## Figures and Tables

**Table 1:** Microsatellite markers

**Locus/marker**	**Location**	**Repeat**	**Nearby located gene**	**Primer sequence**
BAT26	2p22–	(A)_26_	hMSH2, intron 5	F-FAM TGACTACTTTTGACTTCAGCC
				R-AACCATTCAACATTTTTAACCC
				
D3S1611	3p24.2–p22	(CA)_14_	hMLH1, intron 12	F-FAM CCCCAAGGCTGCACTT
				R-AGCTGAGACTACAGGCATTTG
				
D5S346	5q22–q23	(CA)_26_	APC, distance 30–70 kb	F-FAM ACTCACTCTAGTGATAAATCG
				R-AGCAGATAAGACAGTATTACTAGTT
				
D9S171	9p21	(CA)_14_	P16	F-FAM GCTAAGTGAACCTCATCTCTGTCT
				R-GAGATCCTATTTTTCTTGGGGC
				
D11S614	11q23	(A)_13_(GAA)_5_	MLL Intron 6	F-FAM CGCTGGTAATCCCAACACTT
				R-ACCTGGGACTACACGCAACT
				
AFMA301WB5	13q14.3	(CA)_14_	Leu1	F-FAM TCAACATCACCTGTATTCAGCC
				R-CGGCCTCCAAACACTAATTT

**Table 2:** Clinical characteristics

**Patients' samples**	**Age (years)**	**Sex**	**Leucocytes count**	**Binet stage**	**Additional malignancies in the past**
CLL 1	71	Female	22 450	B	None
CLL 2	62	Male	16 730	B	None
CLL 3	63	Female	18 680	A	None
CLL 4	75	Male	139 900	B	None
CLL 5	74	Female	19 000	A	Basal cell carcinoma of skin
CLL 6	72	Female	27 300	A	None
CLL 7	54	Female	63 800	A	Basal cell carcinoma of skin
CLL 8	74	Male	30 000	A	Basal cell carcinoma of skin
CLL 9	69	Female	31 300	A	None
CLL 10	72	Male	23 270	A	None
CLL 11	79	Male	73 300	B	Squamous cell papilloma
CLL 12	73	Male	46 760	B	Adenocarcinoma of prostate, Tubular adenoma of colon × 3
CLL 13	74	Male	81 810	C	None
CLL 14	81	Male	46 180	C	Adenocarcinoma of stomach
CLL 15	68	Male	80 180	C	Basal cell carcinoma of skin × 6, Squamous cell carcinoma of skin
CLL 16	59	Male	34 000	A	None
CLL 17	68	Female	24 130	A	None
CLL 18	74	Female	19 000	A	None
CLL 19	62	Female	24 000	A	None
CLL 20	79	Male	24 500	A	None
CLL 21	86	Male	37 000	B	Adenocarcinoma of colon
CLL 22	68	Male	12 170	A	None
CLL 23	70	Male	19 000	A	None
CLL 24	53	Male	61 240	A	None
CLL 25	45	Female	479 000	C	None
CLL 26	82	Male	20 000	A	None
CLL 27	76	Male	157 600	C	None

**Table 3:** Microsatellite analysis in the study group

**Patients' samples**	**Leu1**	**MLL**	**APC**	**MSH2**	**P16**	**MLH1**
CLL 1	Stable	Stable	Stable	Stable	MSI	Stable
CLL 2	Stable	Stable	Stable	Stable	Stable	Stable
CLL 3	Stable	Stable	Stable	Stable	Stable	Stable
CLL 4	Stable	Stable	Stable	Stable	Stable	Stable
**CLL 5**	**MSI**	**MSI**	**LOH**	Stable	**MSI**	Stable
CLL 6	Stable	Stable	Stable	Stable	Stable	Stable
CLL 7	Stable	Stable	Stable	Stable	Stable	Stable
CLL 8	Stable	Stable	Stable	Stable	Stable	Stable
CLL 9	Stable	Stable	Stable	Stable	Stable	Stable
CLL 10	Stable	Stable	Stable	Stable	Stable	Stable
**CLL 11**	**LOH**	Stable	Stable	Stable	Stable	Stable
**CLL 12**	Stable	**MSI**	Stable	Stable	Stable	Stable
CLL 13	Stable	Stable	Stable	Stable	Stable	Stable
**CLL 14**	**MSI**	**MSI**	**MSI**	**MSI**	Stable	**MSI**
**CLL 15**	**MSI**	**MSI**	**MSI**	**MSI**	Stable	**MSI**
**CLL 16**	Stable	Stable	Stable	Stable	Stable	**LOH**
**CLL 17**	Stable	Stable	Stable	Stable	Stable	**MSI**
CLL 18	Stable	Stable	Stable	Stable	Stable	Stable
**CLL 19**	**LOH**	**MSI**	Stable	Stable	Stable	Stable
CLL 20	Stable	Stable	Stable	Stable	Stable	Stable
**CLL 21**	Stable	Stable	Stable	MSI	Stable	**LOH**
**CLL 22**	Stable	Stable	Stable	Stable	Stable	**LOH**
**CLL 23**	Stable	Stable	Stable	Stable	Stable	**LOH**
**CLL 24**	Stable	**MSI**	Stable	Stable	Stable	**MSI**
CLL 25	Stable	Stable	Stable	Stable	MSI	Stable
CLL 26	Stable	Stable	Stable	Stable	Stable	Stable
**CLL 27**	Stable	Stable	**MSI**	**LOH**	Stable	**LOH**

Bold is used for patients' samples with RER+/MSI in more than 30% of examined loci.
